# Experimental Research on Flexural Mechanical Properties of Ultrahigh Strength Concrete Filled Steel Tubes

**DOI:** 10.3390/ma15155262

**Published:** 2022-07-29

**Authors:** Xiaojun Zhou, Yulin Zhan, Tingmin Mou, Zhilun Li

**Affiliations:** 1 School of Civil Engineering, Southwest Jiaotong University, Chengdu 610031, China; xjzhouedu@163.com (X.Z.); lizhi-lun@my.swjtu.edu.cn (Z.L.); 2 Sichuan Provincial Highway Planning, Survey and Design Research Institute Co., Ltd., Chengdu 610041, China; moutm@vip.sina.com; 3 School of Civil Engineering, Architecture and Environment, Xihua University, Chengdu 610039, China; 4 Institute of Civil Engineering Materials, Southwest Jiaotong University, Chengdu 610031, China

**Keywords:** ultrahigh strength concrete, concrete filled steel tube, flexural bearing capacity, failure mode

## Abstract

Based on the project of the Guansheng Qujiang Bridge, the flexural mechanical properties of an ultrahigh strength concrete filled steel tube (UHSCFST) were discussed. A total of six UHSCFST beam specimens were tested, and the cube strength (*f_cu_*) of the core concrete reached 80.3–115.2 MPa. The effects of concrete strength on flexural bearing capacity, deformation characteristics, and failure modes of UHSCFST specimens were discussed. Test results showed that the bending failure modes of UHSCFST specimens were the same as those of ordinary ones. The failure of UHSCFST specimens was attributed to excessive deflection, and local buckling occurred in the compression zone. Moreover, the bending capacity of the specimens did not decrease, even if they had yielded. Although ultrahigh strength concrete was poured, all of the specimens displayed outstanding bending ductility. The main function of core concrete was to provide radial restraint for the steel tube to avoid premature buckling. When the steel content of the specimen section was constant, the strength increases of core concrete had a slight impact on the bending failure mode, bearing capacity and ductility of UHSCFST specimen. The research results can deepen the understanding of the mechanical behaviors of the UHSCFST composite truss structure.

## 1. Introduction

Concrete filled steel tube (CFST) is a composite structural material formed by pouring concrete into a steel tube. According to its mechanical characteristics, CFST is suitable for an axial compression member. As a flexural member, its advantages cannot be highlighted [1,2]. There are few single CFST flexural member in practical projects, but it is common that CFST suffered bending moment [2,3,4]. For example, the chords of CFST composite truss beams and truss arches, which are widely used in engineering, are mostly in the state of compression bending or tension bending stress [3,4]. Therefore, it is necessary to research the flexural mechanical properties of the CFST member, which will help to understand the working performance of CFST under complex stress conditions. 

It is generally agreed that the support of core concrete within the tube has a positive effect on the mechanical properties of CFST under bending, and its bearing capacity and ductility are significantly improved compared with empty steel tubes [4,5,6,7,8,9,10]. In order to analyze the influence of the filling material strength on the mechanical properties of the flexural member, Kang et al. [11] poured ordinary concrete (*f_ck_* = 27 MPa) and aerated mortar (*f_ck_* = 8 MPa) into the steel tube respectively. It was found that the flexural bearing capacity and ductility of the specimens filled with ordinary concrete were higher than those of the specimens that filled with aerated mortar, and the flexural bearing capacity of the former was about 30% higher than that of the latter. Arivalagan et al. [12] studied the influence of core concrete strength (*f_ck_* = 0.88–32.3 MPa) on the mechanical properties of CFST specimens, and indicated that the flexural bearing capacity of the specimens increased with the enhancement of core concrete strength. However, Al-Obaidi [13] believed that core concrete strength (*f_ck_* = 25–45 MPa) had little effect on the flexural bearing capacity of CFST composite structural members. Faxing [14,15] and Chen [16] also obtained similar conclusions on the flexural mechanical properties of high-strength concrete (*f_cu_* = 46–77 MPa)-filled steel tubes. What about if ultra-high strength concrete (>C80) is used to fill the steel tube? Is there any difference in flexural failure mode and ductility between UHSCFST members and ordinary CFST members? There are almost no relevant reports at present, and these issues need to be addressed.

In China, several reinforced concrete arch bridges have been built, the main arch skeletons of which were composed of UHSCFST trusses [3,4], and C80–C100 concrete was poured into the chord to improve the bearing capacity of the composite truss structure, reduce the construction steps of reinforced concrete outside the truss, and shorten the construction period. Based on the Guansheng Qujiang Bridge, this paper discussed the influence of core concrete strength on the flexural bearing capacity, deformation characteristics, and failure modes of UHSCFST members through model tests, and explored the cross-section strain distribution and development law of UHSCFST beams during the bending process. The test results clarify the flexural mechanical properties of UHSCFST members, and promote the understanding of the mechanical behavior of UHSCFST composite trusses.

This paper is organized in five sections. Section 2 gives a brief background about the project of Guansheng Qujiang bridge built with UHSCFST. In Section 3, the experimental program and the material performance of UHSCFST are introduced. Test progress and results are described and analyzed in Section 4. Finally, a summary and conclusions are given in Section 5. The abbreviations and notations of this manuscript are listed in Table 1. 

## 2. Project Overview

**Figure 1:**
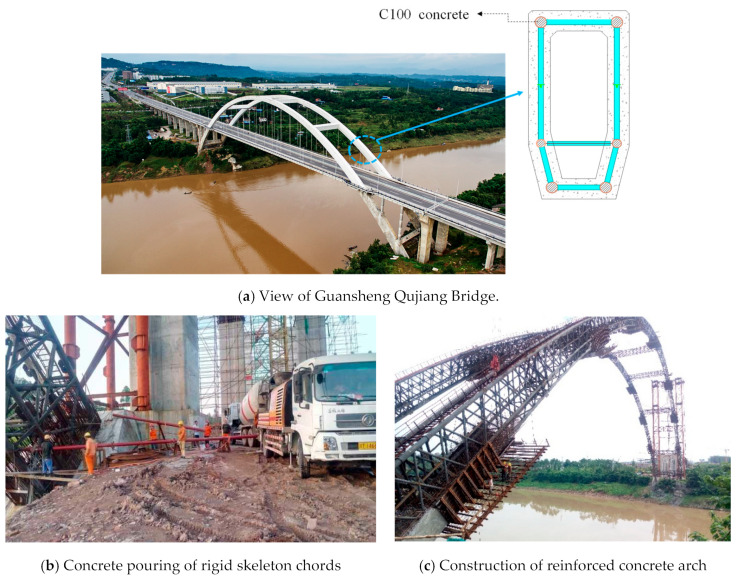
Guansheng Qujiang Bridge, and the steel tube in the rigid skeleton filled with C100 concrete.

Guansheng Qujiang Bridge is a 320 m reinforced concrete half-through arch bridge located in Guang’an, Sichuan Province, China. The main arch construction technology of reinforced concrete arch bridge mainly adopts a cantilever casting method, rigid skeleton arch forming method, etc. In the past, the strength grade of concrete poured in the rigid skeleton main pipe did not exceed C80, and its bearing capacity was limited. Therefore, when the reinforced concrete of the main arch is constructed on the rigid skeleton, it needs to be divided into eight rings, which is a complicated process and takes a long time. However, pouring C100 concrete in the rigid skeleton main tube can obviously improve its bearing capacity, and only two rings are needed for the main arch reinforced concrete [17]. As shown in Figure 1, the main arch ring of the bridge adopts stiff skeleton arch forming technology, which means to use rigid skeleton as the support for the reinforcement binding and concrete pouring of the reinforced concrete arch ring. The rigid skeleton was an UHSCFST composite structure truss, and the webs of the truss were equipped with upper, middle, and lower chords. C100 ultrahigh strength self-compacting micro-expansion concrete was poured into the chords, and C50 self-compacting concrete was used in the reinforced concrete arch [17]. 

## 3. Test Design

### 3.1. Specimen Design and Production

(1) Specimen design

**Figure 2:**
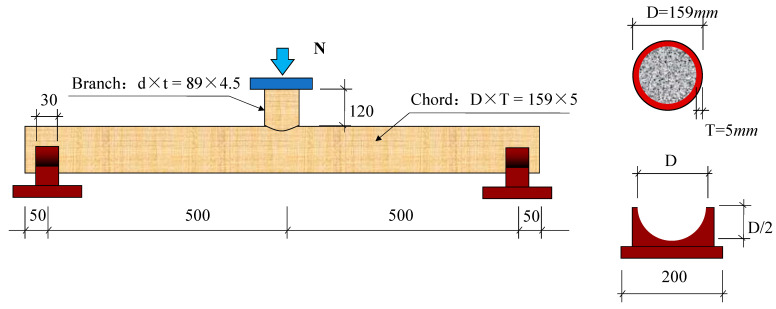
UHSCFST bending specimens (mm).

As shown in Figure 2, in order to investigate the flexural mechanical properties of UHSCFST, a branch tube was welded to the main tube, and the three-point bending test was performed on the main tube by applying load on the branch tube, with the semi-circular arc support utilized. The outer diameter and thickness of main tube is D × T = 159 × 5 mm, and that of the branch tube is d × t = 89 × 4.5 mm. The length of the main tube is 1100 mm, and the height of the branch tube is 120 mm. All specimens were divided into 3 scenarios according to strength of concrete that filled in main tube, each scenario including 2 specimens, and the detailed parameters are shown in Table 2. The “steel content” refers to the ratio of section area of the steel tube to that of the concrete in the tube. Currently, the section steel content of CFST members mainly ranges from 10% to 20% [3,10], and in order to better figure out the influence of ultrahigh strength concrete, the CFST members with lower steel content were utilized. The calculated bearing capacity and bearing moment were calculated according to the Chinese standard DB51/T2598-2019.

(2) Specimen production

The main and branch tubes were cut into the designed length, and welded together according to the intersection line. The rust and debris on the inner wall of the steel tube were removed, and the pipe wall was wetted with water. After that, the main tubes were placed vertically to fill concrete. When the pipe is full, the top concrete should be smoothed and sealed with plastic film. The specimens were leveled after 7 days. Then, the branch tubes were filled with mortar, and the end faces were smoothed and sealed for curing similarly.

### 3.2. Material Performance

#### 3.2.1. Steel Tube

The main tubes and branch tubes were all made of Q345 steel. The mechanical properties of the steel tubes are shown in Table 3.

#### 3.2.2. Concrete

C60, C80, and C100 Concrete were used to fill the main tubes of different specimens respectively. The raw materials of concrete were the same as Guangshen Qujiang Bridge. The mix proportions and key properties are listed in Table 4.

It should be noted that the mix proportion of C100 concrete is completely consistent with the actual project. Polycarboxylate superplasticizer was added when the mixing the concrete. Polycarboxylate superplasticizer was added when the mixing the concrete. The solid content of water-reducing agent was 50%, and the water-reducing rate was 63%. After the dry mixture was evenly mixed for 10–15 s, the water-reducing agent and water were added and stirred for another 120 s. The lump and slump flow of the mixture were 255 mm and 640 mm, respectively, showing satisfactory cohesiveness, wrapping, and fluidity. The expansive agent was a composite high-energy expansive agent consisting of calcium oxide, calcium sulphoaluminate, and magnesium oxide [18]. All specimens were naturally cured in conventional indoor environment without special curing methods. The mechanical properties of concrete are listed in Table 5. The concrete cube strength (*f_cu_*) reached 115.2 MPa at the age of 28 d, which met the requirements of engineering design.

### 3.3. Test Scheme

(1) Test equipment and measuring points

**Figure 3:**
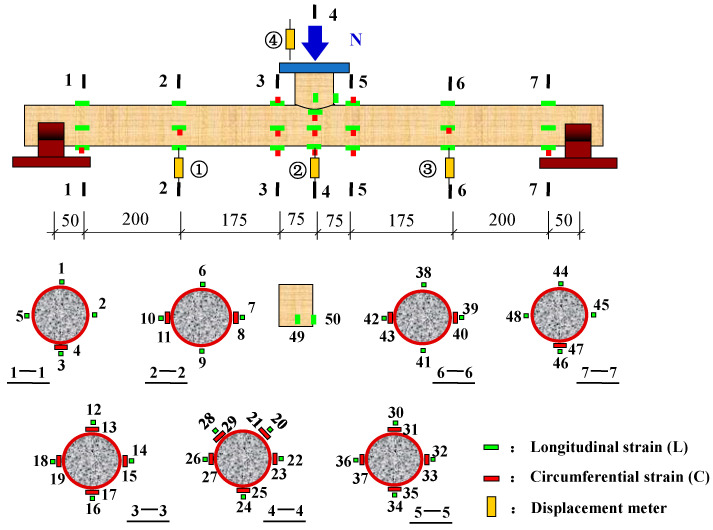
Layout of strain gauges and displacement meters.

**Figure 4:**
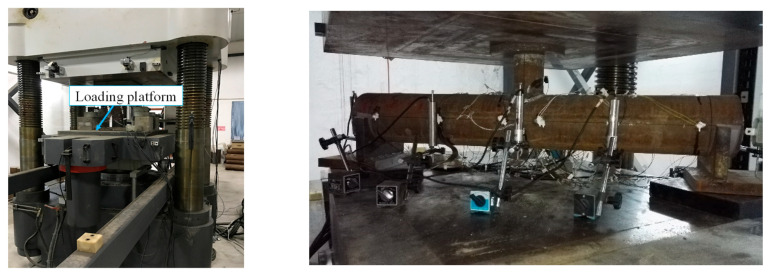
Layout of loading platform.

In order to analyze the strain distribution and development of different sections during the loading process, strain measuring points were arranged in the following positions: the midspan section of main tube (section 4-4), section 3-3, and section 5-5 near the branch tube weld, section 1-1 and section 7-7, which were 20 mm away from the edge of the support, and the 1/4 span section (section 2-2, section 6-6). Meanwhile, another two strain measuring points (No. 49 and 50 strain gauges) were arranged at the bottom of the branch tube to observe whether the branch tube yields before the main tube. There were 50 strain gauges in total, as shown in Figure 3. No. 2 and No. 4 displacement meters were arranged at the top and bottom of the midspan of main tube respectively in order to measure the deformation difference between the upper and lower sections of the main tube. No. 1 and No. 3 displacement meters were arranged at the 1/4 span of the main tube to test the deflection distribution along the length direction of the main tube. All tests were carried out on a 500 t hydraulic servo pressure testing machine, as shown in Figure 4.

(2) Loading plan

Firstly, the load is applied in stages by force control. Each load level is taken as 1/10 of predicted ultimate load. When obvious nonlinear characteristics appear on the load-midspan deflection curve, the load level is reduced to 1/15 of predicted ultimate load. After loading, it should be held for 2 min to observe deformation of the specimen. If yield stage comes, it will be converted to displacement control and loaded slowly and continuously. While one of the following situations occurs, the loading can be stopped and then unloaded: the ratio of deflection to span is close to 1/25 (the midspan vertical displacement is about 45 mm); main tube cracking; main tube collapsed seriously; branch tube buckling.

## 4. Test Process and Test Result Analysis

### 4.1. Loading Process and Failure Mode

**Figure 5:**
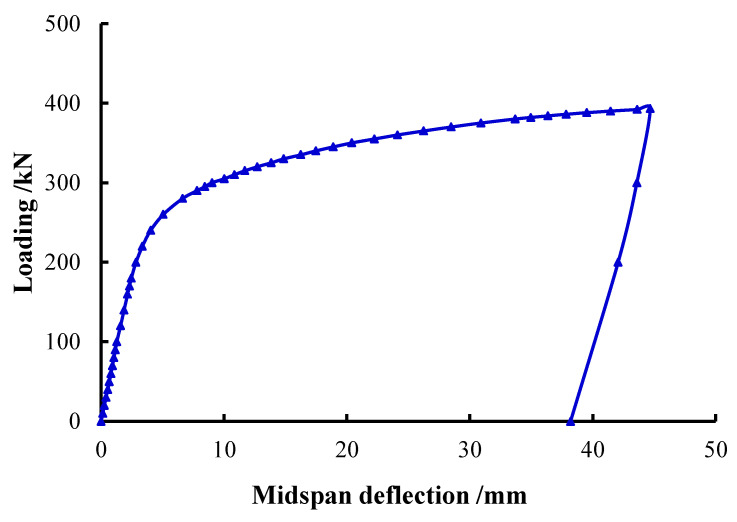
Typical load–midspan deflection curve.

**Figure 6:**
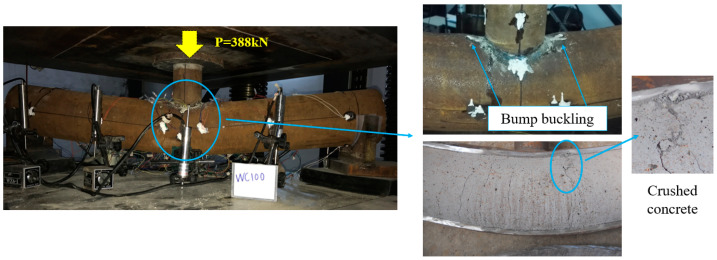
Typical flexural failure mode of UHSCFST specimen.

**Figure 7:**
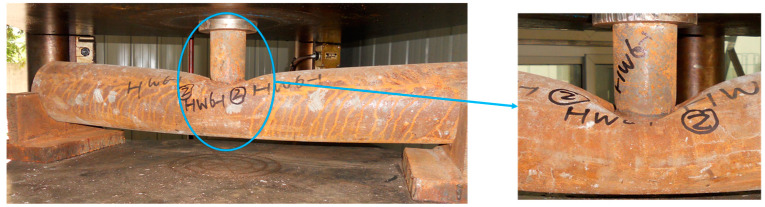
Typical failure mode of hollow steel tubes under bending [19].

At the initial stage of loading, the load increased in proportion to the midspan deflection (Figure 5). When the load went up to nearly 30% of the ultimate load, the load-midspan deflection curve turned slightly due to the concrete cracking in the tensile zone. Subsequently, the load and the midspan deflection continued to increase linearly, and the surface of main tube was basically unchanged. Until the loading was close to 90% of the ultimate load, a cracking sound of core concrete could be heard. Meanwhile, the top surface of main tube in the loading area peeled and the color became darker. Then load growth slowed down, yet the deflection growth accelerated. After that, the concrete cracking sound occurred constantly, the load continued to climb slowly, and the add rate of deflection became stable. Meanwhile, the bending deformation of main tube was gradually obvious, and bulging occurred in the loading area. Finally, the machine was stopped to unload for the large midspan vertical displacement of the specimen. The bending failure characteristics of specimens were different from those of empty steel tubes. The UHSCFST specimens showed overall bending deformation and only slight local buckling in the compression zone (Figure 6), which is similar to that of ordinary CFST specimens [19]. After cutting the steel tube, it was found that the concrete at the bulge was crushed, and the concrete cracks in the tensile zone were equidistant and extended beyond the neutral axis, which is close to the crack distribution of the ideally reinforced beam. However, local collapse buckling was the main failure mode of the hollow steel tubes (Figure 7), and the bending deformation was small.

### 4.2. Load-Deformation Analysis

**Figure 8:**
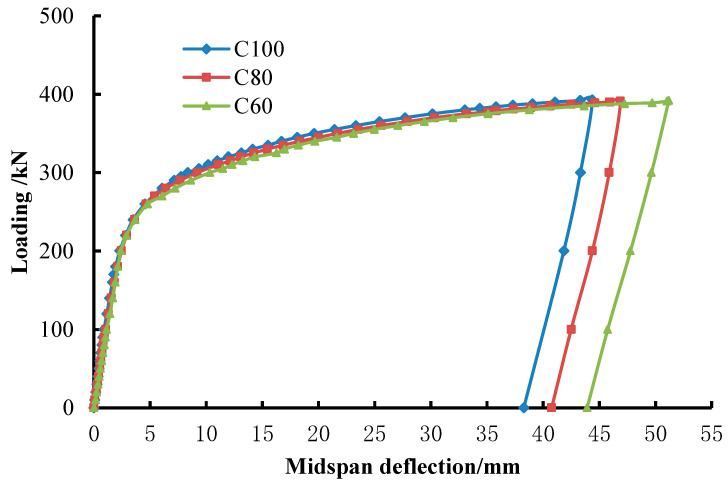
Comparison of three different specimens’ P–ω curves.

**Figure 9:**
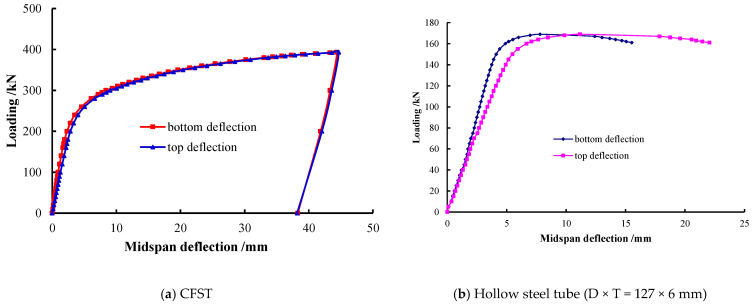
Comparison of deflection at the top and bottom of sections.

The typical load–midspan deformation curve (P–ω curve) of each specimen is shown in Figure 8, and the top and bottom deformation difference of midspan section of CFST and hollow steel tube specimens is compared in Figure 9. The P–ω curves of specimens with C60, C80, and C100 concrete were very similar. They overlapped in the elastic stage, which means that their initial stiffness was close. The curves climbed slowly when the specimens entered the yield state, and the curve of C100 UHSCFST was slightly higher than that of C60 CFST and C80 UHSCFST in the smooth part. It illustrates that the strength of concrete in steel tube has little influence on the flexural stiffness and flexural deformation of CFST. Besides, in Figure 9a, the deflection development of the top and bottom surface of the midspan section of CFST specimen is basically synchronous, which makes it clear that the cross-section kept good integrity and there were few or no in-plane compression deformations. However, as shown in Figure 9b, the deformation of the top surface of the hollow steel tube section was obviously faster than that of the bottom surface [16]. Such behaviors were mainly due to the fact that concrete supports the steel tube to prevent its collapse and buckling, enhances the stiffness of the combined section, changes the shape of the CFST specimen's bending failure, and improves the bending deformation capacity of the specimen effectively [20]. In addition, although the core concrete strengths were very high, midspan deflection increased rapidly and bearing capacity increased slowly after specimens yielding [21]. There were no descending segments in the P–ω curves, and all the CFST specimens had excellent flexural ductility [22].

**Figure 10:**
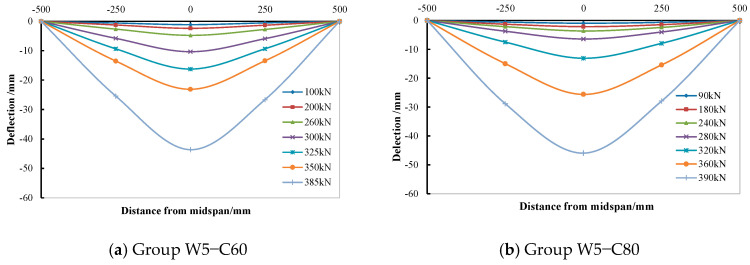

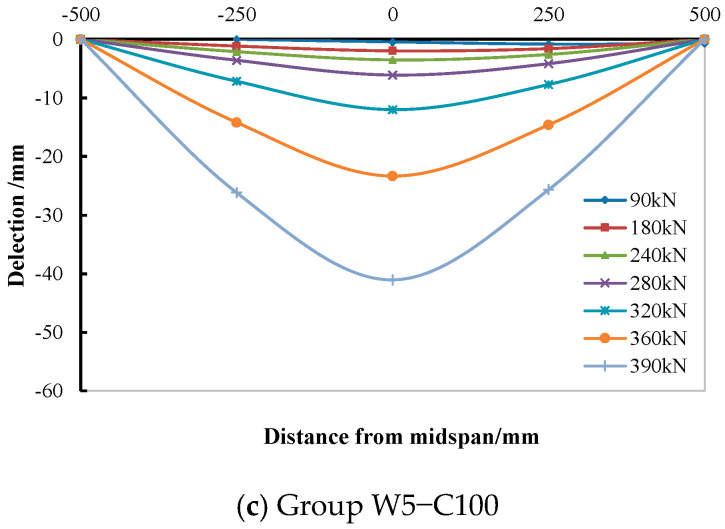
Deflection along the specimen length.

The distribution and development of deflection along the specimen length under different loads are shown in Figure 10. The vertical deflections grew gradually with the load increase. Moreover, the deflection distribution was symmetrical, deflection at midspan was the largest, and that at both ends was small. The deflection curves basically conformed to the stable sine half wave curves, which reflect the overall quality of bending deformation characteristics.

### 4.3. Section Strain Distribution and Development Analysis

The section strain distribution of the three types of CFST specimens along the length was symmetrical, which has nothing to do with the core concrete strength. Therefore, the half span specimen of C100 UHSCFST was taken as the representative to elaborate the section strain distribution and development of the specimens.

**Figure 11:**
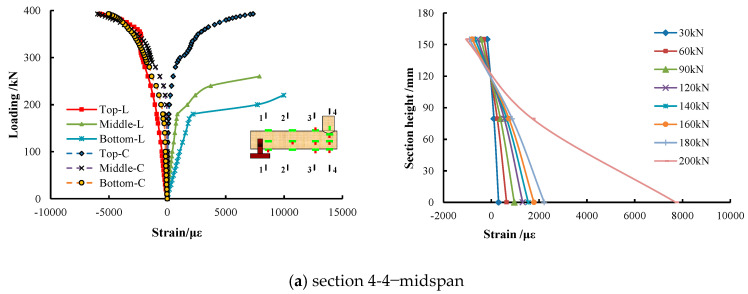

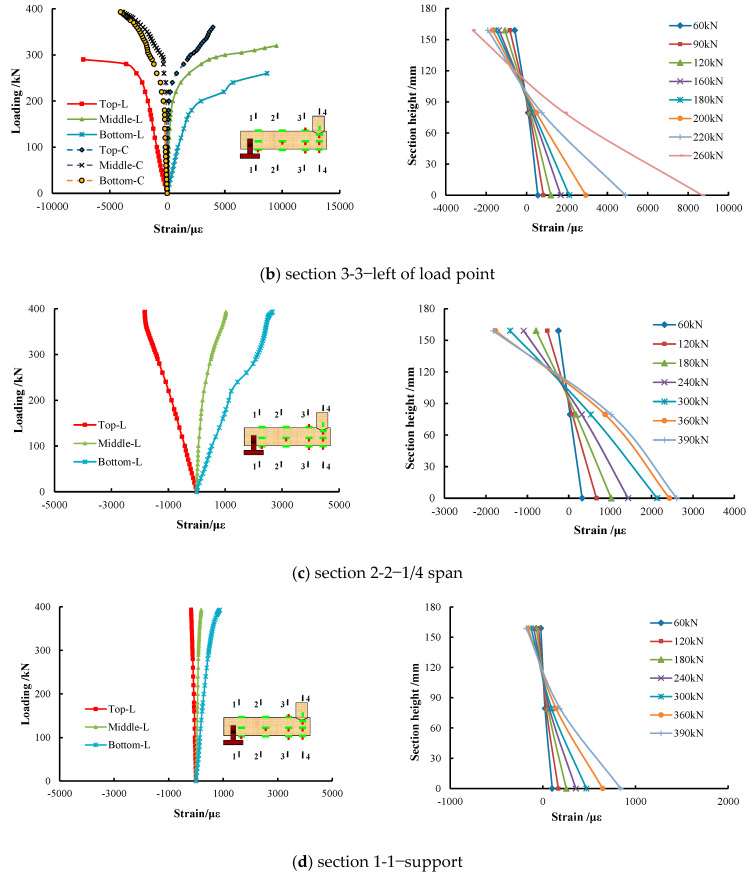
Strain development law of the section of W5-C100-2.

As shown in Figure 11, before yielding and the strain distribution along the height of each section were basically in accordance with the plane section assumption. During the test, the strains of section 1-1 and section 2-2 were small, and almost all of them were elastic strain. Yet, the plastic strains of section 4-4 and section 3-3 were obvious. Section 4-4 is located in the middle of the span, and the longitudinal tensile strain at its bottom reached yield first, while the compressive strain at its top was still in the elastic strain stage and grew slowly. When the load increased to 60% of the ultimate load, tensile strain at its bottom developed rapidly, which led to the failure of the strain gauge and the rapid upward movement of the neutral axis. Then, the bottom of the adjacent section 3-3 was pulled into yield, and the compressive strain at its top was in the elastic strain stage. Tensile strain at the bottom of section 3-3 climbed rapidly as the load increased consecutively, and the strain gauge failed quickly. In the meantime, its top compressive strain also increased dramatically and entered the elastic–plastic strain stage. Since the top strain measurement points of section 3-3 and section 4-4 were respectively located at the crown and saddle areas of the intersecting line of branch tube and main tube, the compressive strain at the top of the former developed faster than that of the latter. When the load reached 80% of the ultimate load, the top of section 3-3 yielded under compression, and it was not until the load reached ultimate load that the top compression strain of section 4-4 began to yield.

### 4.4. Discussion on Bearing Capacity and Calculation Method

The measured loads are shown in Table 4. Ultimate loads were taken as the loads when the ratio of midspan section deflection to span reached 1/50, because the strains at the specimens’ bottoms and tops in the midspan region entered the yield stage at that moment, and after that the load rose slowly and deflection rose rapidly. The proportional ultimate loads of CFST specimens with C60, C80, and C100 concrete were basically the same, and the ultimate load increased slightly with the enhancement of concrete strength. Compared with group W5-C80 and group W5-C60, the flexural bearing capacity of the specimens of group W5-C100 improved by only 0.7% and 2.9%, respectively. Obviously, flexural bearing capacity of UHSCFST specimens had little effect on core concrete strength, which is consistent with the conclusions about ordinary CFST in the literature [12,13,14,15,16,17,18,19]. When the CFST specimen was subjected to bending, both the steel tube and core concrete in the compression zone were under pressure. While the radial deformation of concrete exceeded that of steel tubes, the concrete was restrained by hoop force from the steel tube. However, different from axial compression members, the hoop force was unevenly distributed along the height of the concrete compression zone, and it mainly concentrated near the top steel tube. On the other hand, the concrete cracks extended upward rapidly when the local steel tube at the bottom of tension zone yielded, the neutral axis moved up from the centroid of the section, and the height of the compression zone concrete was small. Because the maximum compressive strain was at the outermost edge of the section, the compressive stress was mainly borne by the steel tube in the compression area, and the bearing effect of concrete on compressive stress was limited. The core concrete mainly provided lateral restraint for the steel tube, avoided partial buckling of the steel tube, and maintained the integrity of the section so as to improve the overall bending capacity and ductility of the specimen.

In Table 6, the calculated flexural bearing capacity of members according to Chinese standard DB51/T2598-2019 and literature [23] is listed, and marked as *M*_uc1_ and *M*_uc2_ respectively. *M_ue_*/*M_uc_*_1_ is about 1.03–1.15, while *M_ue_*/*M_uc_*_2_ is about 0.97–1.06. It can be seen that the results calculated by literature [23] are more consistent with the experimental results. However, both the two calculated flexural bearing capacity increased with the enhancement of concrete strength, which is different from the test results. Generally, the flexural bearing capacities of UHSCFST specimens were higher than that of empty steel tubes. The main reason is that under the support of concrete in the tube, the integrity of the section of CFST was maintained, and the flexural rigidity of the section was further improved. The support tended to be stable when the core concrete strength exceeded a certain value, and then the flexural capacity of CFST would mainly depend on the steel content of the section, and this fact was not considered in the calculation methods proposed by related codes or research [14,15]. Therefore, the reasonable calculation method for the flexural bearing capacity of UHSCFST should be further explored.

## 5. Conclusions

According to the experiment of this paper, it is not difficult to draw the follow conclusions:

(1) The main failure mode of UHSCFST specimen was the overall bending failure, with slight local buckling in the compression zone, and the integrity of the section was maintained well.
(2) The bending failure mode of UHSCFST members resembled that of ordinary CFST, and core concrete strength had little influence on the bending failure mode of UHSCFST.
(3) UHSCFST had high flexural ductility. After the specimen yielded, the bearing capacity was enhanced slowly with the increase of deflection. The load value presented no reduction until the specimen was destroyed.
(4) The increase of core strength had little effect on the flexural bearing capacity of UHSCFST. The main function of core concrete is to support the steel tube wall, and avoid its local collapse and improve the bending stiffness of composite section. However, this effect tends to be stable with the enhancement of core concrete strength, which should be taken into account in establishing the calculation method of UHSCFST bending capacity.

## Figures and Tables

**Table 1:** List of abbreviations and notations.

Abbreviation and Notation	
UHCFST	Ultrahigh strength concrete filled steel tube
CFST	Concrete filled steel tube
*f_cu_*	Concrete cube strength
*f_ck_*	Concrete prism strength
*E_c_*	Elastic modulus of concrete
*P_u_^e^*	Proportional ultimate load
*P_u_^e^*	Ultimate load
*M_uc_*	Test moment
*M_uc_*	Calculated moment

**Table 2:** List of flexural test components.

ID	Specimen Size (D × t × L)/mm	Steel Content	Steel Type	Concrete Grade	Calculated Bearing Capacity/kN	Calculated Bearing Moment/kN·m
W5-C60-1	159 × 5 × 1100	13.87%	Q345	C60	296.8	74.2
W5-C60-2	159 × 5 × 1100	13.87%	Q345	C60	296.8	74.2
W5-C80-1	159 × 5 × 1100	13.87%	Q345	C80	316.4	79.1
W5-C80-2	159 × 5 × 1100	13.87%	Q345	C80	316.4	79.1
W5-C100-1	159 × 5 × 1100	13.87%	Q345	C100	339.6	84.9
W5-C100-2	159 × 5 × 1100	13.87%	Q345	C100	339.6	84.9

**Table 3:** Mechanical properties of the steel tubes.

Type	Size of the Pipe D × t/mm	Steel Style	Yield Strength /MPa	Tensile Strength /MPa	Elastic Modulus (×10^5^)/MPa
Main tubes	159 × 5	Q345	426	585	1.98
Branch tubes	89 × 4.5	Q345	412	553	2.01

**Table 4:** Concrete mix proportions.

Grade of Concrete	Mix Proportion/kg/m^3^							
	Cement	Fly Ash Microspheres	Silica Fume	Expansion Agent	Sand	Gravel	Water	Water Reducer
C100	480	115	70	40	715	1075	127	8.78
C80	410	95	40	30	755	1080	145	4.72
C60	360	70	20	20	765	1095	150	2.12

**Table 5:** Mechanical properties of the concrete.

Grade of Concrete	Slump Flow/mm	*f_cu_*/MPa	*f_ck_*/MPa	*E_c_*/MPa
C100	640	115.2	67.8	48,200
C80	630	95.9	62.0	46,400
C60	600	80.3	57.9	44,800

**Table 6:** Test results of the specimens.

Specimens	Test Loads /kN		Test Moments M_ue_ /kN·m	DB51/T M_uc1_/kN·m	Literature [23] M_uc2_/kN·m	M_ue_/M_uc1_	M_ue_/M_uc2_
	Proportional Ultimate Loads *P_u_^e^*	Ultimate Loads *P_u_*					
W5-C60-1	210	340	85.0	74.2	80.4	1.15	1.06
W5-C60-2	210	340					
W5-C80-1	210	350	86.9	79.1	85.1	1.10	1.02
W5-C80-2	210	345					
W5-C100-1	210	350	87.5	84.9	90.3	1.03	0.97
W5-C100-2	210	350					

## Data Availability

The datasets generated during and/or analyzed during the current study are available from the corresponding author on reasonable request.
